# Digital Remote Assessment of Motor and Speech Changes in Amyotrophic Lateral Sclerosis: Longitudinal Observational Study

**DOI:** 10.2196/85142

**Published:** 2026-07-30

**Authors:** Katherine M Burke, Roland Brown, Narghes Calcagno, Anoopum S Gupta, Krzysztof Z Gajos, Zoe Scheier, Alison Clark, Amrita Iyer, Max P Higgins, Mackenzie Keegan, Timothy B Royse, Evan Remington, Kathryn P Connaghan, Stephen A Johnson, Sheena Chew, M Kelley Erb, James D Berry

**Affiliations:** 1 Neurological Clinical Research Institute and Sean M. Healey and AMG Center for ALS, Department of Neurology, Massachusetts General Hospital, 165 Cambridge Street, 6th Floor, Boston, MA, 02114, United States, 1 6177261844, 1 6177247290; 2 School of Health and Rehabilitation Sciences, MGH Institute of Health Professions, Boston, MA, United States; 3 Biogen, Inc., Cambridge, MA, United States; 4 Department of Neurology and Laboratory of Neuroscience, IRCCS Istituto Auxologico Italiano, Milan, Italy; 5 Department of Neurology, Massachusetts General Hospital and Harvard Medical School, Boston, MA, United States; 6 Department of Computer Science, Harvard John A. Paulson School of Engineering and Applied Sciences, Harvard University, Cambridge, MA, United States; 7 Speech and Social Interaction Lab, MGH Institute of Health Professions, Boston, MA, United States; 8 Department of Neurology, Mayo Clinic, Scottsdale, AZ, United States

**Keywords:** amyotrophic lateral sclerosis, digital health, remote monitoring, motor function, speech

## Abstract

**Background:**

Amyotrophic lateral sclerosis (ALS) is a neurodegenerative disease with an active trial landscape that relies on the sensitivity of selected clinical trial endpoints. Traditional clinical outcome assessments perform well in trials but lack strong psychometric properties and may not detect small but clinically meaningful disease progression. Digital health technologies offer a promising alternative for tracking ALS disease progression.

**Objective:**

This study assessed the feasibility of remote digital monitoring in ALS using a comprehensive battery of prescribed home-based assessments via a smartphone, a wearable device, and a computer-based mouse-clicking task.

**Methods:**

Participants completed weekly remote assessments, including motor and speech tasks via a smartphone app and a computer mouse-clicking task for 24 weeks. They also participated in 3 remote telephone visits in weeks 1, 13, and 25. Reliability, minimal detectable change, and correlations with self-reported ALS Functional Rating Scale–Revised subdomain scores were calculated for 8 features across the speech, fine motor, and gross motor smartphone app tasks and for all 32 features from the computer mouse-clicking task. Sensitivity to longitudinal change was assessed for the 8 smartphone-derived features and for a representative subset of 8 computer mouse-clicking features.

**Results:**

Forty-two participants (19 with ALS and 23 controls) completed 10,237 smartphone assessments and 459 computer mouse-clicking sessions. Baseline discriminative models differentiated ALS from controls with AUC values of 0.75‐0.92. Digital measures correlated strongly with self-reported ALS Functional Rating Scale–Revised subdomain scores. Both participants with ALS and controls demonstrated improvement in fine motor and speech measures, with the exception of nondominant-hand pegboard performance, which declined in the ALS group. Improvements were smaller in participants with ALS, leading to increasing group differences over time, although only one feature showed a statistically significant separation over the 24 weeks. Gait and balance performance declined in both groups, with greater but nonsignificant separation observed for balance measures.

**Conclusions:**

These findings support the feasibility of digital remote assessments in ALS, demonstrate the ability to discriminate between ALS and controls based on certain features collected from speech, fine, and gross motor tasks, and in some cases, quantify functional decline over time. Further research is necessary to explore the natural history of these features longitudinally in larger cohorts of participants with ALS over extended periods to enable their potential integration into clinical trials.

## Introduction

Amyotrophic lateral sclerosis (ALS) is a neurodegenerative disorder that primarily affects motor neurons and leads to progressive muscle weakness and disability [1,2]. US Department of Health and Human Services Food and Drug Administration (FDA)–approved disease-modifying medications for ALS have a modest benefit [3], and thus development of more effective therapies remains a core goal for the field. The successful identification of new therapies in trials will depend heavily on the existence of reliable and sensitive outcome measures. Traditionally, in-person clinical assessments, such as the ALS Functional Rating Scale–Revised (ALSFRS-R) [4], have been used as endpoints and measures of patients’ functional ability. The ALSFRS-R performs well in many regards. However, it is not a patient-reported outcome, so it relies on examiner interpretation of patient responses, introducing subjectivity and interrater variability [5-7]. The scale is ordinal rather than linearly weighted, limiting its sensitivity to detect small but clinically meaningful changes in function across disease progression [8]. Its administration requires a trained clinician, creating a burden related to staff training and clinic time and ultimately limiting its frequency and feasibility in routine care. Given these challenges, there is a need for more sensitive and objective measures to supplement the ALSFRS-R as a trial endpoint.

Digital health technologies (DHTs), such as smartphone apps and wearable devices, offer novel tools and platforms for obtaining highly quantifiable, sensitive, and reliable data about movement, speech, cognition, and gait [9]. DHTs have shown promise in quantifying disease progression across neurological diseases, including Alzheimer disease [10-14], Parkinson disease [15-18], Huntington disease [19], multiple sclerosis [20], and spinal muscular atrophy [21,22]. They are recognized by the FDA as a valuable means for remote data acquisition, supporting efforts to decentralize and diversify clinical trials [23].

In ALS, previous work has demonstrated the feasibility and utility of DHTs for both passive and active remote disease monitoring. DHTs enable the collection of health-related data through both passive and active methods. Passive data collection involves the use of sensors, such as wearable devices, to collect data during everyday activities. Active data collection requires participants to perform specific activities for the purpose of data collection. Tasks for active data collection might include electronic patient-reported outcomes (ePROs) or data collected from wearable sensors during specific task-based assessments, such as audio recordings or timed walking tasks [24]. Passive data collection typically requires extended continuous wear periods due to variability in free-living activities, while active data collection is completed during prescribed tasks, which may require motivation and effort and therefore may be subject to learning effects. The self-reported ALSFRS-R (ALSFRS-RSE), a digital self-assessment of the ALSFRS-R, is feasible, informative, and highly correlated with traditional clinic-based ALSFRS-R administration, supporting its use as an ePRO [25-27].

Beyond the ALSFRS-RSE, there is growing evidence for the use of DHTs to quantify disease progression in ALS [28-34]. DHTs have been used to quantify changes in function using accelerometry for both passive and active data collection of overall activity levels [30,35], gait [36-38], upper-extremity movement [39-43], as well as audio recordings of speech [44-46] and cough [47]. These data suggest that DHTs may have the potential to expedite clinical trials by providing voluminous, objective, and quantifiable data across multiple ALS-relevant domains.

This study evaluated the feasibility of remote, longitudinal digital monitoring of ALS using a comprehensive battery of active, home-based assessments comprised of speech recordings and upper and lower extremity motor tasks delivered via a smartphone-based research app, a wearable device, and a web-based computer program. The performance characteristics of selected features from each motor and speech task were examined to assess baseline differences between participants with ALS and controls and the sensitivity of features to longitudinal change.

## Methods

### Ethical Considerations

The study was approved by the Mass General Brigham (MGB) Institutional Review Board (IRB) prior to initiation (Protocol #2018P002712). All participants provided informed consent prior to the initiation of any study procedures, and data were collected and stored in compliance with MGB policies and regulations and state and national laws. Data collection, management, and security were reviewed by the MGB Information Security Office prior to study initiation.

### Study Design

**Figure 1.**
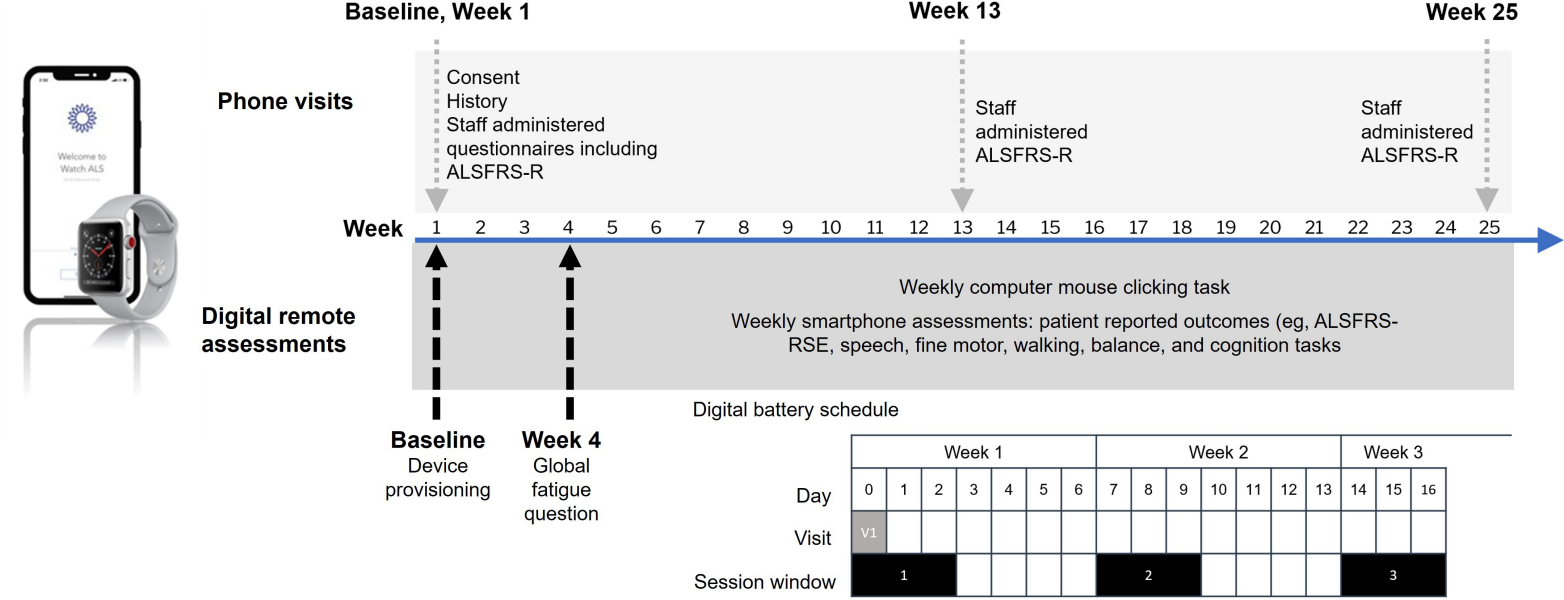
Study design and digital remote assessment schedule. ALSFRS-R: Amyotrophic Lateral Sclerosis Functional Rating Scale–Revised; ALSFRS-RSE: self-reported Amyotrophic Lateral Sclerosis Functional Rating Scale–Revised.

This single-center, longitudinal, observational study was designed to evaluate a comprehensive battery of digital assessments of neurological function in both participants with ALS and healthy controls (HCs). The study was fully remote, with 3 virtual visits with clinic staff and weekly digital assessments completed independently by participants over a 24-week period (Figure 1).

Participants were recruited from the Massachusetts General Hospital (MGH) ALS Clinics, where individuals with ALS are followed clinically. IRB-approved emails were sent out via the ALS clinic email distribution list to people who opted in to receive clinic and research updates. All participants were aged at least 18 years. Participants with ALS met the El Escorial Criteria [48] for possible, probable lab-supported, probable, or definite ALS. Control participants did not carry a diagnosis of ALS or any other neurological condition expected to impact their performance on the digital assessments and had no first-degree relatives with known genetic forms of ALS. HCs were recruited to approximately age-match with the participants with ALS.

### Demographic and Clinical Measures

Baseline clinical history, including time since symptom onset, time since diagnosis, diagnostic criteria, site of onset, fatigue levels on the Neurological Fatigue Index for Motor Neuron Diseases (NFI-MND) [49], and current medications, were obtained by phone interview and through a review of medical records. Additionally, during phone calls with study staff at baseline (week 1), week 13, and week 25 (±7 days), participants completed questionnaires, including the ALSFRS-R [4], reviewed any adverse events related to the study, and provided feedback on both the mobile app and its implementation on the smartphone devices, including usability and technical issues.

### Digital Remote Assessments

On a weekly basis, participants completed both a mobile assessment customized for the study and a point-and-click computer mouse task remotely [50,51]. The mobile assessment was completed on a provisioned Apple smartphone (iPhone 8+ or 12 Pro; OS versions 12.1‐15.1) and smartwatch (Apple Watch 4 or 5; OS versions 5.1.2‐8.0) via the WatchALS smartphone app built from the BrainBaseline platform (Clinical ink). The assessment in the WatchALS app was customized to capture patient-reported outcomes of mood, fatigue, and symptoms related to changes in ALS, as well as active tasks to capture limb and speech data (Table 1). The sampling rate during active limb motor tasks was 100 Hz.

Speaking tasks were recorded by the internal microphone of the smartphone. They included 15 seconds of syllable sequence repetition (/pa ta ka/) and reading the Bamboo Passage [52,53]. Participants were prompted to repeat the tasks if there was excessive background noise or they could choose to do so for any other reason. The total number of correctly produced syllables during syllable repetition was divided by 15 seconds to calculate the articulation rate. Speaking rate, calculated as words per second, was calculated for the Bamboo Passage reading task.

Fine motor tasks consisted of an alternating finger-tapping task and a digital grooved pegboard task, each completed with their dominant and nondominant hands as they were able. In the finger-tapping task, they used their index and middle fingers to alternately tap 2 circles on the screen as quickly as possible for 20 seconds, and the total alternating taps were the primary outcome. In the digital pegboard task, they placed virtual pegs into designated positions on the screen as quickly as possible, the total number of completions was captured, and a standardized adjusted score was the primary outcome.

**Table 1.** Smartphone app digital tasks and feature descriptions.

Domain and assessment	Sensor / modality	Frequency	Features/scores	Meaning of higher values
Current symptoms				
Symptom questionnaire	Survey, Likert	Weekly	Item scores	Worse
Global function				
ALSFRS-RSE^a^	Survey	Weekly	Total score	Better
Motor speech function				
Syllable repetition of the sequence /pa ta ka/	Smartphone internal microphone	Weekly	Articulation rate	Better
Passage reading (Bamboo Passage)	Smartphone internal microphone	Weekly	Speaking rate	Better
Upper extremity motor function				
Alternating finger tapping	Smartphone touchscreen	Weekly	Total alternating taps	Better
Digital pegboard	Smartphone touchscreen	Weekly	Adjusted score	Better
Walking and balance				
60-second walk test	Accelerometer	Weekly	Gait speed	Better
30-second balance test	Accelerometer	Weekly	Box-Cox sway area	Worse

a ALSFRS-RSE: self-reported Amyotrophic Lateral Sclerosis Functional Rating Scale–Revised.

Gross motor function was assessed with a 60-second walking activity performed at the participants’ usual pace and a 30-second static balance activity. For the walking task, they walked back and forth for 60 seconds at their normal walking speed in an area that was at least 10 feet long. Gait speed was the primary outcome and calculated as the distance covered divided by the time spent walking. During the static balance task, participants stood still with their arms by their sides and feet shoulder-width apart for 30 seconds and sway area, calculated as the total movement of the phone during the task, was the primary outcome. They wore the phone in a belt across their lower backs, and it was paired with the watch such that synchronous sensor data could be obtained from the wrist and lower back.

Activities on the smartphone became available at midnight on the first day of the week, and participants had a 3-day window to complete the tasks. Automated reminders were provided on the study phone when the activities opened each week and from study coordinators at MGH as needed. Once the participant began a session, all activities for that window were to be completed within an hour. Data from completed activities were saved even if the full battery couldn’t be finished in an hour. The remainder of the tasks in these sessions were recorded as incomplete attempts. If the participant quit any assessment early, no data were recorded for that assessment, and it was recorded as an incomplete attempt. The participants were given the option to skip any of the tasks and would indicate the reason for skipping the task. The deidentified raw data from the watch and phone were automatically transferred from participants’ devices to a secure cloud where they could be accessed by study staff for analysis.

The computer mouse clicking task was used separately from the WatchALS smartphone app and was completed on a computer using an internet browser. Participants entered their participant code, gender, age, handedness, and answered questions about computer usage, including which hand they would use for the task. Participants were asked to log in each week to complete the task. There were no automated reminders for the computer mouse clicking task. Instructions were provided on the site, and participants were asked to click on a dot (the target) when it appeared on the screen. The first time a participant accessed the computer mouse clicking task, there was a practice session to identify the minimum target size that each participant was able to click on reliably. During the task, the targets were set to be at least as large as the minimum target size identified in that initial practice session. The main task comprised 8 blocks, 9 trials each, with the first click used to put the mouse cursor in a controlled starting position for the subsequent movement. Complete computer mouse movement trajectories were collected as participants clicked on targets on the screen. This task has previously been validated in a supervised clinical setting for patients with ataxias and parkinsonism [50], as well as in a home setting for children with ataxia-telangiectasia [51], and adults with ataxias [54]. The deidentified web-based computer task data are stored on a commercial server hosted by DreamHost [50,51].

### Signal Processing and Feature Selection

#### Smartphone App

The key features analyzed for each of the fine motor, speech, and gross motor tasks are presented in Table 1. The following digital features collected from the smartphone tasks were selected a priori for use in all discriminatory and longitudinal analyses, including dominant and nondominant hand total taps from the alternating finger-tapping task, dominant and nondominant hand adjusted scores from the digital pegboard task, articulation rate from the syllable repetition task, speaking rate from the passage reading task, gait speed from the walking task, and total sway area from the static stance balance task.

For the syllable repetition task, acoustic signals were processed using a method adapted from Rong [55]. A Hilbert transform was used to extract acoustic envelopes of the audio signal. Peaks in the envelope were identified with peak detection and corresponded to individual syllables. The acoustic signals were segmented into voiced and unvoiced periods using a voice activity detection algorithm [56]. Articulation rate was calculated by dividing syllables identified during voiced periods by 15 seconds.

For the Bamboo Passage, speaking rate was estimated using an automated transcription approach (AWS_transcribe). Audio files were transcribed using AWS Transcribe (Amazon Web Services), and the transcription was scored against the Bamboo Passage target text using *Levenshtein* distance edit operations [57] that identify correctly spoken words from the text, missing words, substitutions, and insertions. The timing of the first and last correctly spoken words or substitutions was identified as the start and end of the passage to avoid initial and final silences. After subtracting the duration of any insertions and immediately following pauses, speaking rate (words per minute) was defined as the passage word length (number of words) divided by the passage duration. The quality of automated transcription for estimating speaking rate was evaluated by comparing the results to those obtained from manually transcribed files (n=60), where we found excellent agreement (intraclass correlation [ICC]>0.97).

For the walking task, participants were allowed to use an assistive device, and they indicated if one was used for the task or not. For each participant, we marked each session as “no assistive device” or “used assistive device.” We found that some participants used devices variably across sessions. This small, exploratory study was designed to evaluate whether accelerometry could be used to quantify gait in individuals using and not using assistive devices. The study was not powered to assess intraparticipant variability associated with intermittent use of gait assistive devices over the 24 weeks. Including mixed sessions (some with and without device use) would have inflated within-participant variability, biased longitudinal estimates of gait speed, and undermined the interpretability of reliability metrics, given the size of this study. Therefore, gait speed analyses were restricted to the condition most frequently used by each participant across the study period. If a participant used an assistive device for most sessions, only sessions completed with an assistive device were included in the gait speed analysis, and sessions completed without an assistive device were excluded, or vice versa. Total sway area was observed to be strongly right-skewed with some extreme outliers. To diminish the effect of these very large values and better satisfy normality and other modeling assumptions, sway area observations were capped at 0.5 cm^2^, followed by a Box-Cox transformation with λ=0.2.

#### Computer Mouse Clicking Task

The feature extraction and analysis for the mouse clicking task have been described in detail previously [50,51]. In short, we could derive 32 features from the movement trajectories of the mouse during this task. These features are representative of the separate components of the task, including initiation time (from target onset to first mouse movement), execution time (from first to last mouse movement), verification time (time spent on the target once the mouse has stopped to the start of the click), and click time (time from mouse click down to mouse click up). The first trial of each block was used to position the mouse and was excluded from the analyses. Pauses were marked whenever there was a break of 100 ms or more for raw mouse movement. The results for each participant were analyzed as age-specific *z*-scores (compared with a normative dataset collected previously from over 200,000 healthy volunteers), separately for each of the 32 features. They were calculated separately for each block of trials and later averaged across the blocks.

### Statistical Analysis

The R statistical software package (version 4.2.0; R Foundation for Statistical Computing) and the Python (Python Software Foundation) programming language were used for data analysis. Demographic, clinical characteristics, and adherence data were summarized with descriptive statistics. Adherence metrics were computed as a proportion of the total expected number of assessments. For the participants with ALS, baseline ALSFRS-R was used to determine an imputed baseline rate of decline (points per month), defined as 48-baseline total score divided by the number of months since symptom onset.

#### Reliability and Measurement Sensitivity

To assess reliability and measurement sensitivity in the digital features, we computed ICC1 and minimum detectable change (MDC) for each feature. Separate estimates were computed for participants with ALS and HC and used the entire longitudinal dataset (rather than a single pair of measurements) to enable greater estimate precision. For each digital measure, a one-way linear random effects model ignoring time with a subject-specific random intercept was used for each group to estimate between- and within-subject variance ($\sigma_{s}^{2}$ and $\sigma_{e}^{2}$, respectively). These quantities were then used to calculate $ICC1=\frac{\sigma_{s}^{2}}{\sigma_{s}^{2}+\sigma_{e}^{2}}$ and $MDC95=1.96*\sigma_{e}*\sqrt{2}$. We additionally computed 95% CIs for ICC1 using the parametric bootstrap. MDC was calculated at the group level using the pooled within-subject variance across participants. This approach yields a single MDC value that represents the minimum detectable change for the measure in this sample, rather than individual-specific MDC estimates.

Because the entire longitudinal dataset was used for computing ICC and MDC quantities, we had concerns about temporal trends unrelated to the measurement properties in question (eg, disease-related decline or learning effects) distorting the estimates. In particular, macro-longitudinal trends could inflate the within-subject variance and downwardly bias ICC. To account for this possibility, we estimated ICC and MDC on “detrended” datasets. These datasets were generated by (1) subtracting the fitted participant-specific generalized additive mixed models (GAMM)–estimated mean trajectories from the raw data values (described below in the longitudinal analysis), followed by (2) adding back in the subject-specific intercept, captured as their baseline performance. This process has the effect of removing high-level temporal trends while retaining both the within- and between-subject variability of interest. An example of this detrending process can be seen in Figure S3 in Multimedia Appendix 1. We observed that, as expected, the detrending process resulted in modest increases in estimated ICCs relative to using the raw data. CIs for ICC did not account for uncertainty in the detrending process, so they may be slightly anticonservative.

#### Baseline Performance

Baseline performance on the 8 features chosen a priori across the speech, fine, and gross motor tasks was summarized using means and SDs for participants with ALS and HC. Differences in means and corresponding 95% CIs were calculated for each feature, and unadjusted *P* values were obtained using independent-samples *t* tests, assuming unequal variances where appropriate. All tests were 2-sided, and statistical significance was evaluated at an alpha level of .05. No adjustment was made for multiple comparisons.

#### Discriminative Models

Logistic regression and random forest models were used to predict group (people with ALS vs HC) using the 8 features chosen a priori across the speech, fine, and gross motor tasks. Due to small sample size, tuning parameters in the random forests were fixed a priori (number of trees=300, max depth=3, variables per node=3). Repeated 10-fold cross-validation—stratified by ALS/HC status to ensure class balance in the folds—was used to evaluate model performance. To understand sensitivity of model performance to measurement variability, AUC estimates with 95% CIs were computed using first (baseline) observation only, second only, third only, and fourth only, along with the averages across the first 2, first 3, and first 4 observations.

#### Correlation With ALSFRS-R

To assess the concurrent validity of the digital measures, Spearman correlations at baseline were calculated between digital features and each subdomain of the ALSFRS-R, including bulbar (questions 1‐3), fine motor (questions 4‐6), gross motor (questions 7‐9), and respiratory (questions 10‐12). For these, 95% CIs were computed using the nonparametric bootstrap.

#### Longitudinal Analysis

Group-specific mean curves were estimated as smooth functions of time using GAMMs, which were preferred over linear mixed models because they allowed for nonlinearity in the longitudinal trajectories. Random effect smooths allow each individual’s estimate to deviate from the mean curves, while simultaneously shrinking individual curves toward the mean curves, similar to traditional random effects. GAMMs were implemented using the gam() function in the *mgcv* R package [58]. Thin plate regression splines (option bs=“tp”) were used to fit the group-specific smooth fixed effects, factor-smooth interactions with a common smoothness parameter were used for the random effect smooths (option bs=“fs”), and restricted maximum likelihood was used for estimation of the smoothing parameters.

For each digital feature, the *emmeans* R package [59] was used to compute contrasts of interest and 95% CIs from the fitted GAMMs, including the mean change from baseline to the end of the study in people with ALS and HC group and the mean between-group difference at baseline and at the end of the study. Statistical tests derived from the GAMMs assess whether longitudinal changes differed between patients with ALS and HCs.

For the smartphone app tasks, the longitudinal analysis focused on the same 8 features evaluated at baseline and in the discriminative models. For the computer mouse clicking task, we present results from 8 representative features selected following visual inspection of the spline models for each of the 32 features. Trajectories over time varied substantially among the full feature set. Given the exploratory nature of this analysis and recognizing that features with high short-term repeatability are not necessarily those most sensitive to disease-related change, the selected features illustrate the diversity of longitudinal feature behavior.

## Results

### Demographics and Clinical Characteristics

A total of 23 HCs and 19 people living with ALS enrolled in this remote study. Demographic information and clinical characteristics are presented in Table 2. The participants with ALS and HCs were similar in age, race, and ethnicity (both groups were predominantly white and non-Hispanic). A higher proportion of males was observed in the ALS group (73.7%), compared with HCs (39.1%). Baseline ALSFRS-R scores for participants with ALS averaged 31.8 (SD 7.9), highlighting the diverse functional status and disease severity represented in the cohort.

**Table 2.** Participant baseline characteristics.

Participants	People with ALS^a^ (n=19)	Healthy controls (n=23)
Demographics		
Age (years), mean (SD)	60.6 (5.6)	58.0 (8.2)
Male (sex), n (%)	14 (73.7)	9 (39.1)
White (race), n (%)	18 (94.7)	21 (91.3)
Site of onset, n (%)		
Lower extremity	9 (47.4	—^b^
Upper extremity	6 (31.6)	—
Bulbar	2 (10.5)	—
Generalized	2 (10.5)	—
Baseline fatigue score on the NFI-MND^c^, mean (SD)		
Total score	12.8 (3.3)	3.6 (4.3)
Energy subscore	8.8 (3.2)	3.1 (3.9)
Weakness subscore	11.5 (2.6)	1.9 (3.0)
Disease duration at time of enrollment (months), mean (SD)	51.9 (34.7)	—
Baseline ALSFRS-R^d^, mean (SD)	31.8 (7.9)	47.3 (0.9)
Baseline bulbar subscore	10.1 (2.9)	12 (0.0)
Baseline fine motor subscore	6.5 (2.9)	11.7 (0.5)
Baseline gross motor subscore	5.7 (2.4)	11.7 (0.6)
Baseline respiratory subscore	9.5 (3.2)	11.8 (0.5)
Baseline ALSFRS-R slope (pts/month), mean (SD)	–0.41 (0.29)	—

a ALS: amyotrophic lateral sclerosis.

b Not applicable

c NFI-MND: Neurological Fatigue Index for Motor Neuron Diseases.

d ALSFRS-R: Amyotrophic Lateral Sclerosis Functional Rating Scale–Revised.

### Digital Assessments: Feasibility and Adherence

#### Smartphone App

**Figure 2.**
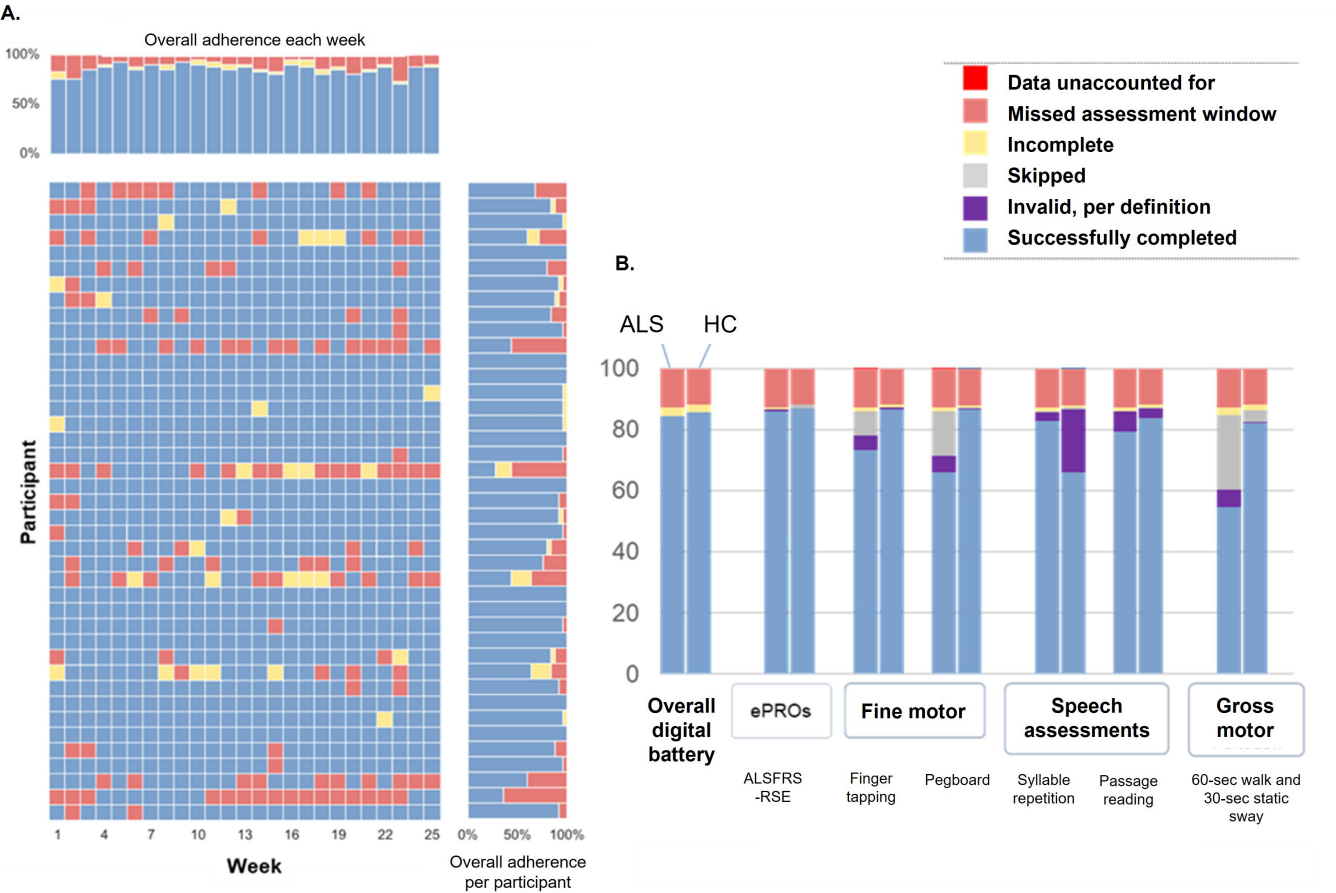
WatchALS adherence data by (**A**) each participant across the study period and (**B**) representative study tasks for participants with ALS and HCs. Assessments were classified as complete, incomplete, or missed, depending on whether all tasks were completed, partially completed, or not initiated. Task completion status was categorized as successfully completed (task completed with valid data), invalid (task completed but features could not be reliably extracted from the raw data), skipped (task intentionally skipped by the participant), incomplete (task initiated but not completed), missed (no tasks initiated during the assessment window), or data unaccounted for (task completed but corresponding raw data files were unavailable). ALS: amyotrophic lateral sclerosis; ePRO: electronic patient-reported outcome; HC: healthy control.

Over the 24-week study period, 10,237 digital assessments were performed across all participants (active data collection), and 6801 hours of continuous sensor data were recorded from participant watches (passive data collection). Participants successfully completed a total of 872 (85.07%) of a possible 1025 assessment batteries within the study smartphone app. Completion rates were comparably high between those with ALS and HC (84.4% and 85.6%, respectively). Adherence data for many of the smartphone tasks were presented in Figure 2A-2B. Notably, participants with ALS tended to skip more of the gross motor tasks compared with HC and relative to other tasks, primarily due to task difficulty. Speech tasks presented challenges in data quality, resulting in a higher rate of invalid or unusable data, primarily due to very low speech volume among some participants or rapid task completion among control participants, which limited the ability of the automated approach to distinguish individual syllables.

#### Computer Mouse Clicking Task

Participants completed 459 computer mouse clicking sessions. Among the 19 participants with ALS, 14 completed at least one session, averaging 15 sessions (1‐23 range) per participant. Of the 23 HCs enrolled, 18 completed at least one session, averaging 14 sessions (1‐25 range) each.

#### Remote Telephone Visits

All participants completed each of the 3 telephone visits with study staff.

### Reliability

#### Smartphone App Tasks

The test-retest reliability, presented by the ICCs for each of the features evaluated, is presented in Table 3. For the smartphone fine motor features, total alternating finger taps from dominant and nondominant hands demonstrated the highest ICCs in both participants with ALS (0.95, 95% CI 0.90‐0.97 and 0.96, 95% CI 0.92‐0.98, respectively) and HCs (0.94, 95% CI 0.89‐0.96 and 0.92, 95% CI 0.85‐0.96, respectively). Among the speech features, speaking rate had the highest ICC in participants with ALS (0.97, 95% CI 0.93‐0.98). Syllable articulation rate also demonstrated high ICC in participants with ALS (0.91, 95% CI 0.82‐0.95) and was the highest ICC for the HC group (0.86, 95% CI 0.76‐0.92). Gait speed showed high reliability for the ALS group (0.93, 95% CI 0.85‐0.96) and moderate reliability for HCs (0.68, 95% CI 0.50‐0.78). Notably, many participants with ALS were either unable to complete the walking task or changed the assistive devices they used during the task throughout the study duration, affecting the reliability of this measure.

The minimal detectable change at 95% confidence level (%MDC95) was calculated to identify the smallest detectable change in each feature for each task that exceeded the threshold of measurement error with 95% confidence, representing the minimal change over time in an individual that can be confidently interpreted as a true change rather than measurement variability (Table 3). As expected, the %MDC95 values for most features were similar between people with ALS and HCs, except for the speaking rate and gait speed. For speaking rate, the %MDC95 was 22.1 words per minute for participants with ALS and 31.3 words per minute for HCs, indicating that a greater change in words per minute is required in HCs to be considered a true change beyond measurement error. A similar pattern was observed for gait speed, where a change of 0.18 meters per second was required for people with ALS, compared with 0.28 meters per second for HCs to exceed the MDC threshold.

**Table 3.** Reliability, minimal detectable change (%MDC95), and correlations with subdomains of the self-reported Amyotrophic Lateral Sclerosis Functional Rating Scale-Revised (ALSFRS-RSE) for task features.

Domain, assessment, and features/scores	ICC^a^ (95% CI)		%MDC95^b^		Correlation coefficients with subdomains of the ALSFRS-R^c^			
	ALS^d^	HC^e^	ALS	HC	Bulbar	Fine motor	Gross motor	Respiratory
Upper extremity motor function								
Alternating finger tapping								
Total alternating taps (dominant hand)	0.95 (0.90 to 0.97)	0.94 (0.89 to 0.96)	35.5	33.8	0.17 (–0.48 to 0.68)	0.74 (0.41 to 0.93)	–0.28 (–0.72 to 0.35)	–0.04 (–0.53 to 0.50)
Total alternating taps (nondominant hand)	0.96 (0.92 to 0.98)	0.92 (0.85 to 0.96)	32.6	36	0.22 (–0.46 to 0.72)	0.71 (0.23 to 0.92)	–0.08 (–0.60 to 0.42)	–0.05 (–0.54 to 0.45)
Digital pegboard								
Adjusted score (dominant hand)	0.73 (0.49 to 0.83)	0.54 (0.35 to 0.67)	12.9	14.5	0.02 (–0.56 to 0.60)	0.71 (0.23 to 0.92)	0.00 (–0.54 to 0.53)	0.23 (–0.38 to 0.72)
Adjusted score (nondominant hand)	0.81 (0.63 to 0.89)	0.51 (0.31 to 0.64)	12.9	14	–0.08 (–0.70 to 0.66)	0.53 (–0.17 to 0.85)	0.21 (–0.48 to 0.79)	0.34 (–0.38 to 0.77)
Motor speech function								
Syllable repetition								
Articulation rate (syllables/s)	0.91 (0.82 to 0.95)	0.86 (0.76 to 0.92)	1.03	1.05	0.51 (0.02 to 0.81)	0.16 (–0.33 to 0.59)	–0.37 (–0.71 to 0.08)	0.36 (–0.14 to 0.70)
Passage reading								
Speaking rate (words/min)	0.97 (0.93 to 0.98)	0.79 (0.64 to 0.86)	22.1	31.3	0.36 (–0.21 to 0.73)	0.21 (–0.38 to 0.60)	–0.07 (–0.56 to 0.47)	0.21 (–0.29 to 0.62)
Walking and balance								
Walking task								
Gait speed (m/s)	0.93 (0.85 to 0.96)	0.68 (0.50 to 0.78)	0.179	0.284	–0.45 (–0.78 to 0.24)	0.26 (–0.49 to 0.71)	0.83 (0.61 to 0.95)	–0.40 (–0.71 to 0.08)
Balance activity								
Box-Cox transformation of sway area (cm^2^)	0.87 (0.72 to 0.92)	0.55 (0.36 to 0.68)	1.51	1.3	0.31 (–0.32 to 0.78)	0.32 (–0.40 to 0.82)	–0.01 (–0.52 to 0.48)	0.23 (–0.37 to 0.70)

a ICC: intraclass correlation.

b %MDC95: minimal detectable change at the 95% confidence level.

c ALSFRS-R: Amyotrophic Lateral Sclerosis Functional Rating Scale–Revised.

d ALS: amyotrophic lateral sclerosis.

e HC: healthy control.

#### Computer Mouse Clicking Task

For the computer mouse clicking task, ICCs across the 32 features varied greatly, ranging from 0.38 to 0.96 in the ALS group and 0.35‐0.78 in HCs, as outlined in Table S1 in Multimedia Appendix 1. The most reliable features for the participants with ALS were movement time (0.96, 95% CI 0.92‐0.98), normalized jerk with pauses (0.94, 95% CI 0.86‐0.97) and without pauses (0.94, 95% CI 0.86‐0.97), execution time with pauses (0.94, 95% CI 0.84‐0.97) and without pauses (0.93, 95% CI 0.83‐0.96), and verification time (0.91, 95% CI 0.81‐0.96). For HCs, the ICCs were lower than those observed in the ALS group, with the highest performing features being movement time (0.76, 95% CI 0.58‐0.85), maximum acceleration (0.70, 95% CI 0.46‐0.81), and verification time (0.78, 95% CI 0.60‐0.87). Additionally, %MDC95 was computed for each of the features and is presented in Table S1 in Multimedia Appendix 1.

#### Baseline Performance

**Figure 3.**
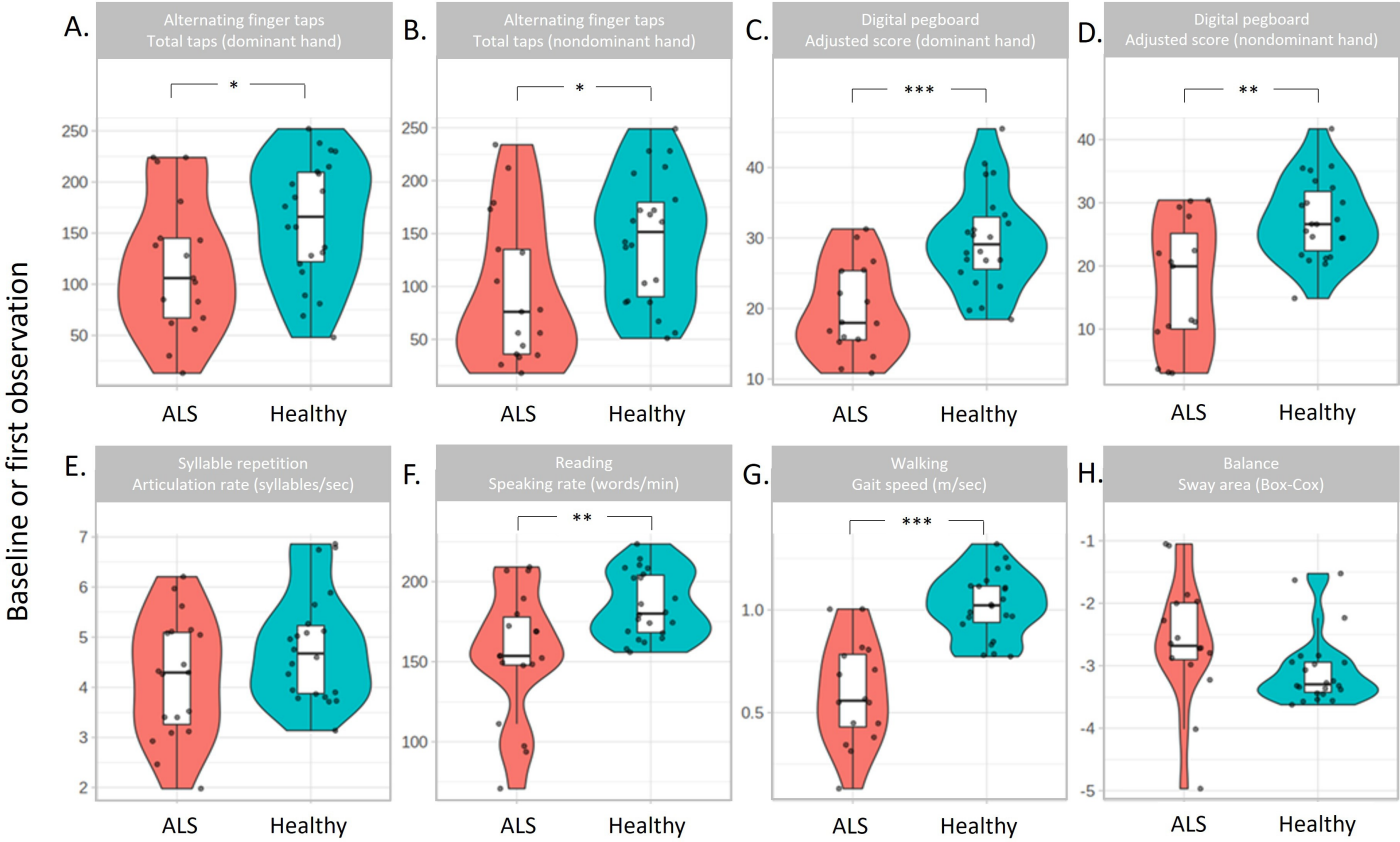
Baseline performance for participants with ALS compared with healthy controls for each of the smartphone assessments: (**A**) dominant hand finger taps, (**B**) nondominant hand finger taps, (**C**) dominant hand digital pegboard task adjusted score, (**D**) nondominant hand digital pegboard task adjusted score, (**E**) articulation rate on the syllable repetition, (**F**) speaking rate on the reading task, (**G**) gait speed on the walking task, and (**H**) Box-Cox transformed sway area for the static stance balance task. Asterisks indicate statistical significance for the difference in means between participants with ALS and HCs: *<0.05, ** <0.01, and ***<0.001 (unadjusted *P* values). ALS: amyotrophic lateral sclerosis.

Baseline performances on each of the smartphone task features were compared between participants with ALS and HC (Figure 3A-3H and Table S2 in Multimedia Appendix 1). Participants with ALS consistently demonstrated lower average performance compared with HCs across multiple domains. Specifically, participants with ALS completed fewer alternating finger taps, placed fewer pegs during the pegboard task, had slower speaking and articulation rates, slower gait speed, and increased sway area on the balance task compared with the HC group, with all these findings except for sway area and articulation rate reaching statistical significance.

#### Discriminative Analyses

**Figure 4.**
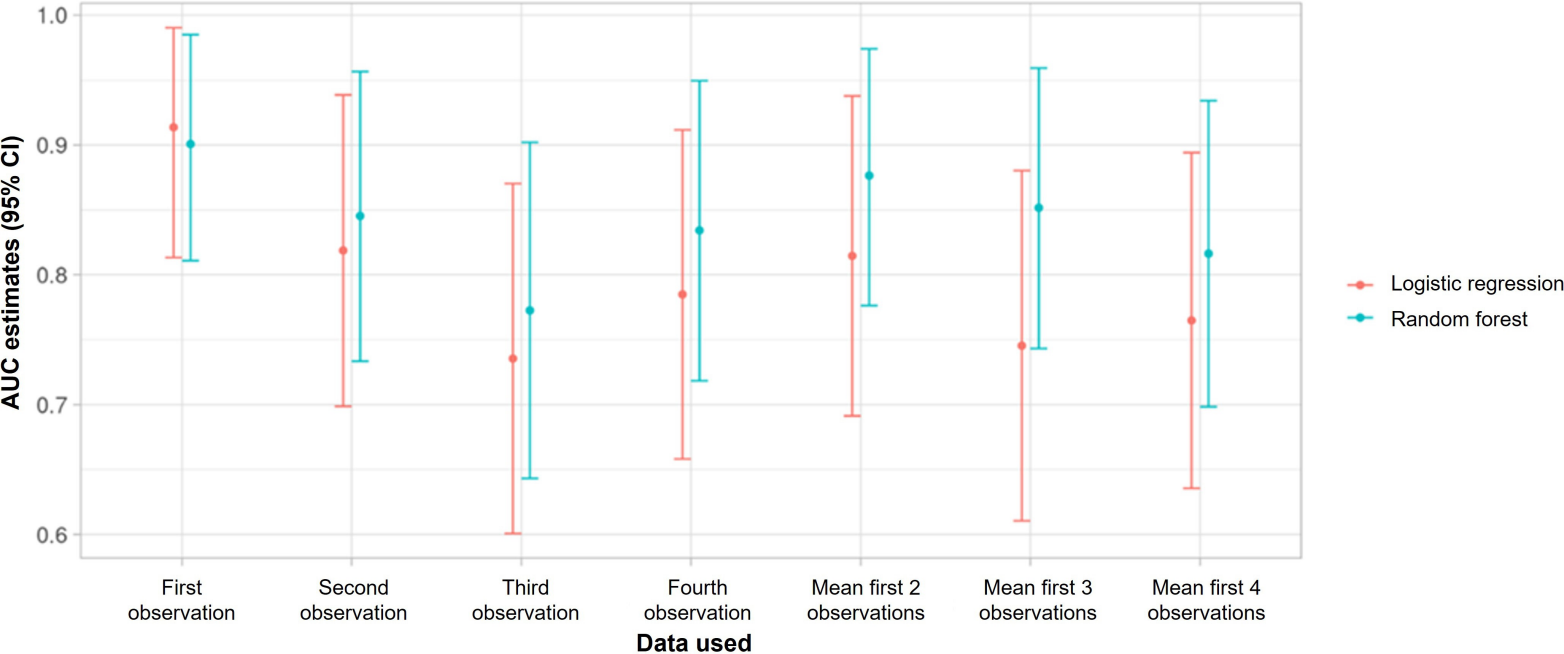
Area under the curve (AUC) estimates for the predictive models for participants with ALS versus healthy controls (HCs) using 8 features from the smartphone assessments (dominant and nondominant hand total taps on alternating finger tapping and adjusted scores on digital pegboard, syllable repetition articulation rate, speaking rate from the reading tasks, gait speed from the walking task, and total sway area from the static stance balance task) at baseline (first observation) and the next 3 observations (second, third, and fourth), as well as for the mean of the first 2 observations, first 3 observations, and first 4 observations using logistic regression and random forest methods. AUC: area under the curve.

AUC estimates predicting ALS versus HC status using 8 features chosen a priori—dominant and nondominant hand total taps on alternating finger tapping and adjusted scores on digital pegboard, syllable repetition articulation rate, speaking rate, gait speed, and total sway area—show high predictive ability with AUC estimates ranging from 0.75 to 0.92 (Figure 4). Model performance is shown separately using these features at baseline, week 1, week 2, week 3, and the average of the first 4 observations. There is considerable variability seen in the predictive performance depending on which observation or sets of observations were used.

#### Correlations With ALSFRS-RSE and Subdomains

Features from each smartphone task generally showed the strongest Spearman correlations with their corresponding ALSFRS-RSE subdomains (Table 3).

Of the smartphone upper extremity features, dominant-hand alternating finger taps demonstrated the strongest correlation with the fine motor subdomain (questions 4‐6) of the ALSFRS-RSE (0.74, 95% CI 0.41‐0.93). Among the speech features, syllable articulation rate was found to have the highest correlation with the bulbar subdomain (questions 1‐3; 0.51, 95% CI 0.02‐0.81). Gait speed was highly correlated with the gross motor subdomain (questions 7‐9; 0.83, 95% CI 0.61-0.95). Representative scatterplots are provided in Figure S1 in Multimedia Appendix 1.

### Longitudinal Analysis

#### Smartphone App

**Figure 5.**
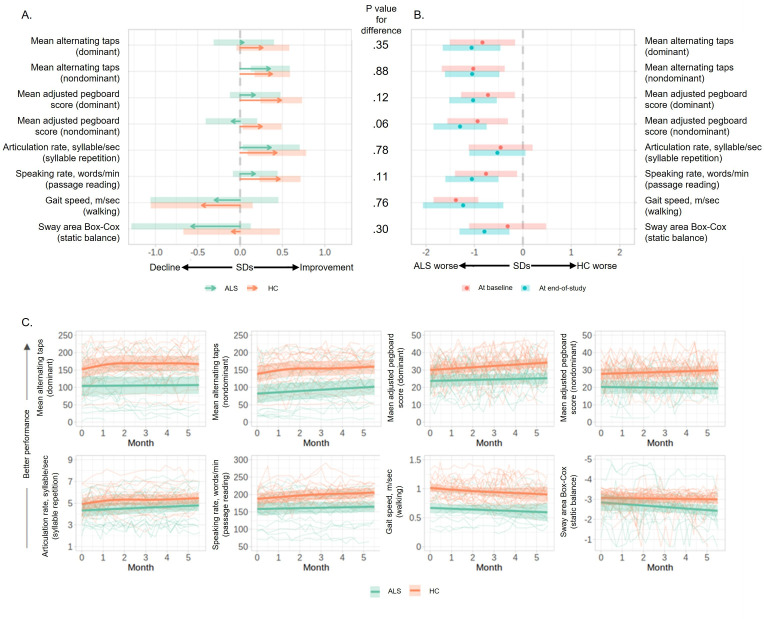
Generalized additive mixed model (GAMM) results assessing longitudinal performance in dominant hand total taps on alternating finger tapping, nondominant hand total taps on alternating finger tapping, dominant hand adjusted score on digital pegboard, nondominant hand adjusted score on digital pegboard, syllable repetition articulation rate, speaking rate from the reading tasks, gait speed from the walking task, and Box-Cox transformed total sway area from the static stance balance task. (**A**) Mean end-of-study changes from baseline for each of the features by ALS/HC status in SD units, computed as contrasts from the GAMM models. Positive value indicates improvement in performance and negative value indicates decline in performance. (**B**) Mean group separation comparing participants with ALS with healthy control (HC) performance at baseline and end-of-study in SD units. Positive values indicate a worse performance in HC and negative values indicate a worse performance in participants with ALS, computed as contrasts from the GAMM models. Higher absolute values indicate greater group separation. *P* values correspond to tests of whether group separation was different at end-of-study versus baseline (red vs blue estimates in B), or equivalently, whether longitudinal change was different between ALS and healthy groups (orange vs green arrows in A). (**C**) Fitted mean curves and 95% confidence bands for ALS and HC groups, overlaid on individual trajectories. Y-axis directionality is such that up indicates better performance.

In the smartphone tasks, both participants with ALS and HC demonstrated improvement over time in the fine motor and speech tasks (Figure 5A). The exception was the nondominant hand digital pegboard mean adjusted score, where participants with ALS showed a decline in function at the end of the study (Figure 5A). Notably, the HC group improved more, widening the performance separation by the end of the study (Figure 5B), perhaps due to ALS blunting the learning effect. This increased mean group difference was most pronounced for the adjusted pegboard scores and speaking rate but did not reach significance in this small sample size.

In gross motor tasks, participants with ALS consistently performed worse than HC, and both groups showed a decline in performance (Figure 5A and B). The difference in sway area widened, as it did for other features. The difference in gait speed between the 2 groups slightly narrowed by the end of the study. Performance variability was observed in both ALS and HC participants throughout the study. This was evident at both the individual participant level and in the aggregated group data (Figure 5C).

#### Computer Mouse Clicking Task

**Figure 6.**
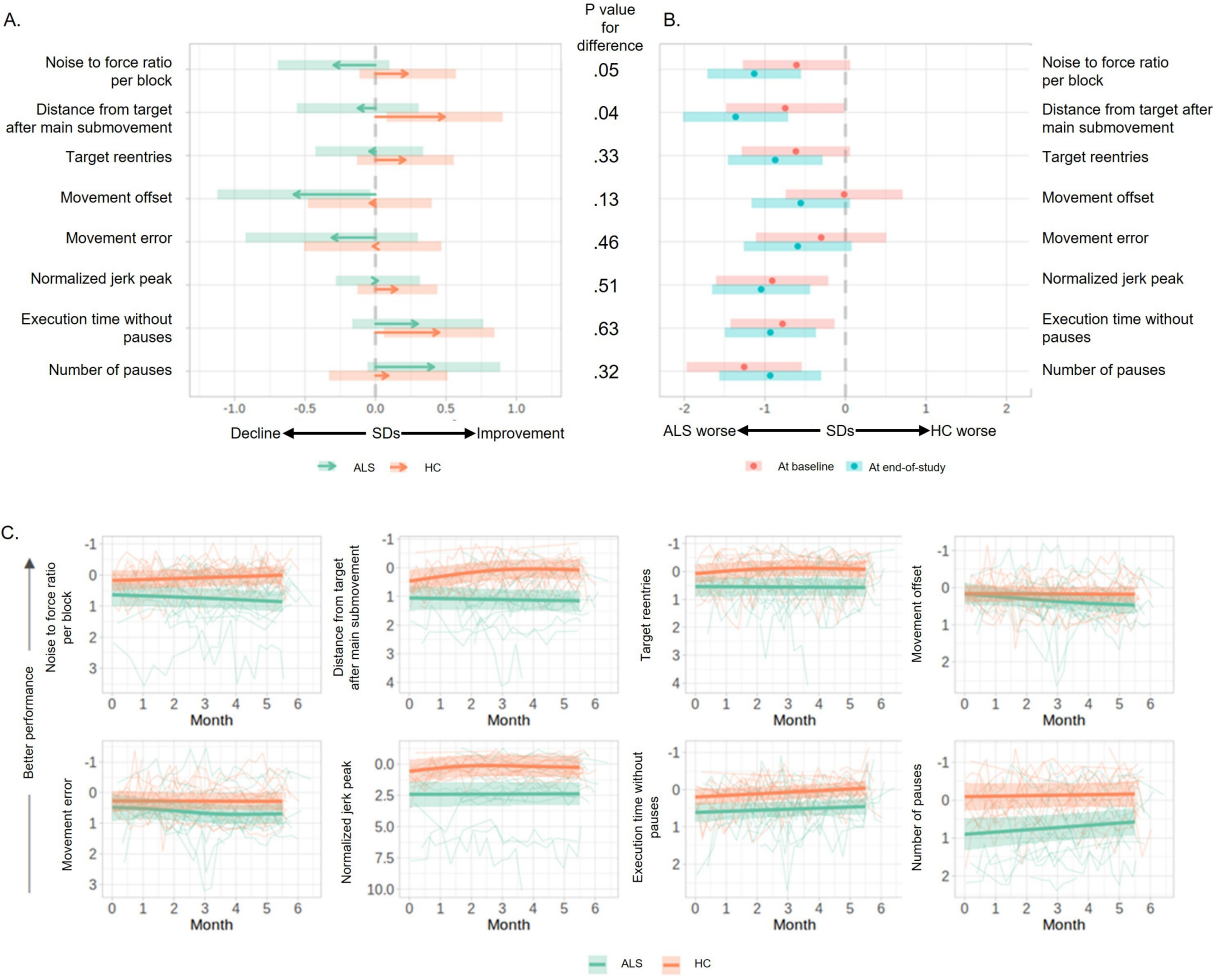
Generalized additive mixed model (GAMM) results assessing longitudinal performance in 8 of the 32 features from the computer mouse clicking task: noise to force ratio per block, distance from target after main submovement, target reentries, movement offset, movement error, normalized jerk peak, execution time without pauses, and number of pauses. (**A**) Mean end-of-study changes from baseline for each of the features by amyotrophic lateral sclerosis (ALS)/healthy control (HC) status in SD units, computed as contrasts from the GAMM models. A positive value indicates improvement in performance, and a negative value indicates a decline in performance. (**B**) Mean group separation comparing participants with ALS with HC performance at baseline and end-of-study in SD units, computed as contrasts from the GAMM models. Positive values indicate a worse performance in HC, and negative values indicate a worse performance in participants with ALS. Higher absolute values indicate greater group separation. *P* values correspond to tests of whether group separation was different at end-of-study versus baseline (red vs blue estimates in B), or equivalently, whether longitudinal change was different between ALS versus healthy groups (orange vs green arrows in A). (**C**) Fitted mean curves and 95% CI bands for ALS and HC groups, overlaid on individual trajectories. ALS: amyotrophic lateral sclerosis.

We selected 8 features from the computer mouse clicking task by choosing representative patterns from the spline models for each of the 32 features (Figure S2 in Multimedia Appendix 1). These 8 features are (1) noise-to-force ratio per block (SD of the distance from the target center at the end of the first submovement divided by the mean maximum acceleration), (2) distance from the target after the main submovement, (3) target reentries (number of times the cursor leaves and re-enters the target before clicking), (4) movement offset (mean distance of the cursor from the task axis), (5) movement error (mean absolute distance of the cursor from the task axis), (6) normalized jerk (movement smoothness adjusted for movement duration and amplitude), (7) execution time excluding pauses, and (8) number of pauses, representing the different patterns seen across all features. Overall, participants with ALS exhibited a decline in performance in 5 of these 8 features (noise-to-force ratio per block, distance from target after main submovement, target reentries, movement offset, and movement error), with improvement in the other 3 (normalized jerk, execution time without pauses, and number of pauses) over study duration (Figure 6A). In contrast, HCs showed improved performance in all but 2 (movement offset and movement error) of the selected features (Figure 6A). Overall, participants with ALS performed worse than HC in each feature at baseline, and the separation from HC increased over study duration in all features, except for number of pauses (Figure 6B). Only distance from the target after the main submovement reached significance in this small sample size. Similar to the smartphone app tasks, performance variability was observed in both ALS and HC participants throughout the study, at both the individual participant level and in the aggregated group data (Figure 6C).

## Discussion

### Principal Findings

In this study, we evaluated the feasibility, reliability, and validity of smartphone- and computer-based motor and speech tasks in individuals with ALS compared with HCs. Our study shows that speech and limb motor features from a comprehensive digital remote assessment can be used to distinguish people living with ALS from HCs and monitor ALS progression. The tasks and features were reliable, discriminatory, well correlated with their respective subdomains on the ALSFRS-RSE, and sensitive to change over time, consistent with previous studies [22,39,60] and adding to the growing evidence supporting the use of DHTs for individual patient monitoring and as clinical outcome assessments in ALS clinical trials.

### Feasibility and Adherence

Collecting digital assessments across multiple impairment domains is feasible using a smartphone app, a wearable device, and a computer app. Even taking incomplete adherence into account, data collection frequency far exceeded what can be captured in-person. Adherence rates surpassed those reported in many previous studies [29,31,60,61], with smartphone tasks showing higher adherence than the computer mouse clicking task, likely due to the greater convenience of smartphone tasks, the absence of a log-in requirement, the automated reminders, and close monitoring by study staff for the smartphone tasks. In addition to the scheduled phone calls, study staff proactively contacted participants who had missed consecutive smartphone assessments, which facilitated early troubleshooting and contributed to the high adherence for these tasks, highlighting the importance of frequent touchpoints between participants and study staff for successful data collection.

### Comparison With Previous Studies

Speech motor function, quantified by articulation rate (syllable repetition) and speaking rate (passage reading), was reliable and moderately correlated with the ALSFRS-RSE bulbar subdomain. However, these correlations were lower than previous work [62] and lower than those observed for fine and gross motor tasks with their respective subdomains. These differences are likely the result of a somewhat restricted population in this study; only 3 participants with ALS scored below 10 on the bulbar subdomain of the ALSFRS-RSE. Importantly, despite this lack of heterogeneity and relatively higher bulbar function in our ALS group, speaking rate significantly distinguished the ALS from the HC group, consistent with previous studies [46].

Consistent with prior studies demonstrating the utility of typing tasks, prescribed upper extremity exercises, and digital finger-tapping tasks [41,42,63,64], the findings in this study further support the utility of fine motor tasks administered via both a smartphone app and a computer web browser. These measures distinguished participants with ALS from HCs, correlated with the fine motor subdomain, and demonstrated sensitivity to longitudinal change, supporting their potential utility as a DHT-derived clinical outcome [65].

Many of the speech and fine motor features improved over time in both groups, but the rate of improvement was slower in participants with ALS, suggesting a blunted learning effect that may reflect underlying disease progression. The widening mean group separation over the course of the study indicates that participants with ALS experienced a decline or lack of learning relative to HC. This underscores the continued need for placebo arms in trials, where randomization and placebo-adjusted treatment effect estimates are essential to mitigate the confounding effects of learning.

Gross motor function, quantified by gait speed and sway during the static balance task, was reliable and significantly distinguished participants with ALS from the HCs. There were outliers in the sway data, which has been seen in previous work in Parkinson disease [66], necessitating adjustments in statistical analysis plans for trials. Gait speed correlated strongly with the ALSFRS-RSE gross motor subdomain, consistent with prior research [67]. While gait speed is a well-established functional measure across broad populations [67-71], our study highlights some of the complexities specific to ALS gait analysis, particularly related to changes in the assistive device use over time and a potential motivational component, as longitudinal analysis demonstrated a decline in performance in both those with ALS and the HCs [72-77]. The size of our study constrained the gait assistive device analyses to a binary variable (device or no device). Future studies could develop more complex analytical approaches, for example, longitudinal analysis of the specific type and timing of assistive device use. While the gross motor tasks (balance and walking) provided valuable insights, there is a clear measurement floor, since participants need to stand for these measurements. Future studies could develop tasks that do not require independent standing to quantify gross motor function.

### Limitations

Despite these promising findings, several limitations should be acknowledged. Given the heterogeneity in ALS presentation, a broad set of tasks spanning multiple impairment domains may be crucial for early diagnosis and precise monitoring across clinical phenotypes. Our predictive models showed promising performance, but given the small sample size, they were influenced by missed or skipped assessments, highlighting the need for larger studies to optimize data collection frequency and refine predictive capabilities. This study was designed as an exploratory, hypothesis-generating investigation to evaluate the initial feasibility and performance of a digital remote assessment in ALS and was not powered to detect group-by-time interactions. Therefore, the findings related to group differences and patterns of change over time should be interpreted as exploratory and intended to inform future, adequately powered studies. Additionally, the study population examined was somewhat restricted, with limited diversity in ALS presentation, particularly in the bulbar subdomain. As a result, larger studies with a more diverse cohort of individuals living with ALS are needed to validate the findings in this study.

Our gait analysis may oversimplify the complexity of gait changes in people with ALS, as assistive device adoption and frequency of device use may influence measures such as gait speed. Further investigation into the impact of assistive device use on digital gait metrics is warranted. We also selected representative metrics to quantify performance on each of the tasks; however, other digital metrics may have performed better in distinguishing between people living with ALS and HCs and may be more sensitive to tracking disease progression over time. Future work should not only validate additional digital metrics but also compare these metrics to understand the value of each.

### Conclusions

Ultimately, this study underscores the potential of DHTs to gather data remotely and frequently and to quantify disease progression in people living with ALS. Most of the features examined here significantly distinguished participants with ALS from HC and demonstrated increased separation over time. There is substantial opportunity to expand this research by exploring additional features and tasks, integrating free-living behavioral data to circumvent learning effects, determining how these tasks might predict meaningful markers in disease progression (eg, functional milestones or event-free survival), and collecting participant feedback to better understand task burden and motivation.

DHTs are unlikely to serve a single universal purpose in ALS; different measures may be optimized for complementary roles, such as early disease detection, clinical phenotyping, or quantifying overall disease progression. These complementary roles underscore the importance of defining the appropriate context of use and validation pathways for each digital measure within the frameworks for digital endpoint development. A comprehensive and participant-centered approach could significantly enhance our ability to track ALS disease progression and accelerate drug development.

## Supplementary material

10.2196/85142 Multimedia Appendix 1 Supplementary tables and figures for the reliability of computer mouse clicking task features, baseline group comparisons, correlations with ALSFRS-RSE scores, spline models, and detrending analyses.

## Abbreviations

%MDC95: minimal detectable change at 95% confidence level
ALS: amyotrophic lateral sclerosis
ALSFRS-R: Amyotrophic Lateral Sclerosis Functional Rating Scale–Revised
ALSFRS-RSE: self-reported Amyotrophic Lateral Sclerosis Functional Rating Scale–Revised
AUC: area under the curve
DHT: digital health technology
ePRO: electronic patient-reported outcome
FDA: US Department of Health and Human Services Food and Drug Administration
GAMM: generalized additive mixed model
HC: healthy control
ICC: intraclass correlation coefficient
IRB: Institutional Review Board
MDC: minimal detectable change
MGB: Mass General Brigham
MGH: Massachusetts General Hospital
NFI-MND: Neurological Fatigue Index for Motor Neuron Diseases
