# Supplementary material for: Digital Remote Assessment of Motor and Speech Changes in Amyotrophic Lateral Sclerosis: Longitudinal Observational Study
Source: JMIR Form Res. 2026 Jul 30;10:e85142. doi: 10.2196/85142 (PMC13423962; doi:10.2196/85142)

**Supplementary Tables and Figures**

Supplementary Table 1. Reliability and MDC for the Features from the Computer Mouse Clicking Task.

Note: Features are described in detail in the supplementary materials for Gajos et al., 2019[50], values were averaged for each block and the Box-Cox transform was applied to make the distribution of the values approximately normal. Bolded values represent the five highest ICCs for each of the two groups.

Supplementary Table 2. Baseline measurement group performance comparisons

| **Measure** | **Healthy Control Mean (SD)** | **Participants with ALS Mean (SD)** | **Difference in Means (95% CI)** | **Unadjusted P-value** |
| --- | --- | --- | --- | --- |
| Alternating Finger Taps Total Taps (Dominant Hand) | 162 (59.4) | 118 (66) | 44 (2.2, 85) | **0.040** |
| Alternating Finger Taps Total Taps (Non-Dominant Hand) | 145 (59.3) | 95.8 (69.4) | 50 (6.7, 93) | **0.025** |
| Digital Pegboard Adjusted Score (Dominant Hand) | 29.7 (7) | 19.8 (6.4) | 9.9 (5.4, 14) | **< 0.001** |
| Digital Pegboard Adjusted Score (Non-Dominant Hand) | 27.4 (6.4) | 17 (10.1) | 10 (4.3, 17) | **0.002** |
| Syllable Repetition Articulation Rate (syllables/sec) | 4.79 (1.1) | 4.18 (1.2) | 0.61 (-0.12, 1.3) | 0.099 |
| Passage Reading  Speaking Rate (words/min) | 185 (20.6) | 154 (39.9) | 31 (9.4, 52) | **0.007** |
| Walking Gait Speed  (m/s) | 1.02 (0.2) | 0.595 (0.2) | 0.43 (0.28, 0.57) | **< 0.001** |
| Balance Sway Area (Box-Cox) | -3.07 (0.5) | -2.61 (1) | -0.46 (-1.03, 0.11) | 0.110 |

Supplementary Figure 1. Correlations of the (a) finger taps with dominant hand and ALSFRS-RSE fine motor subdomain score, (b) adjusted score on the digital pegboard for the dominant hand and the ALSFRS-RSE fine motor subdomain score, (c) gait speed on the walking task and the ALSFRS-RSE gross motor subdomain score, and (d) mean articulation rate on the syllable repetition and the ALSFRS-RSE bulbar subdomain score.


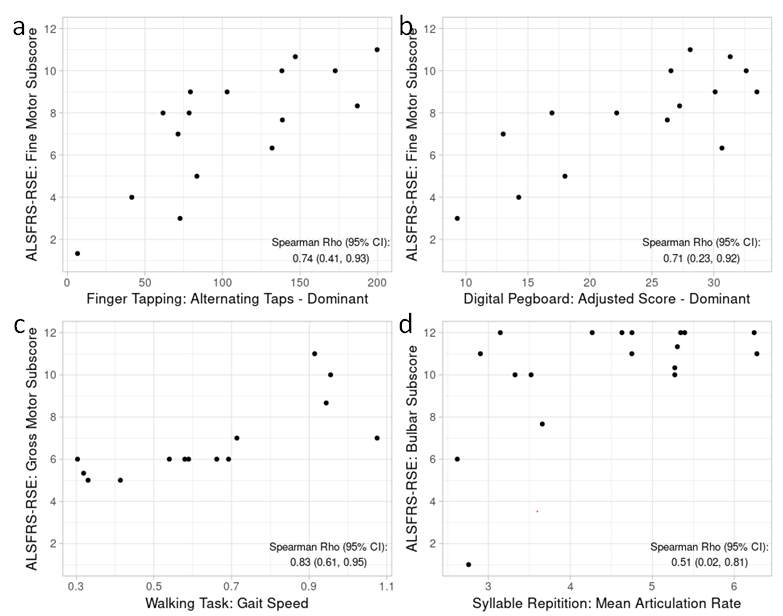


Supplementary Figure 2. Spline models for the 32 features captured by the computer mouse clicking task for ALS and healthy control (HC) participants.


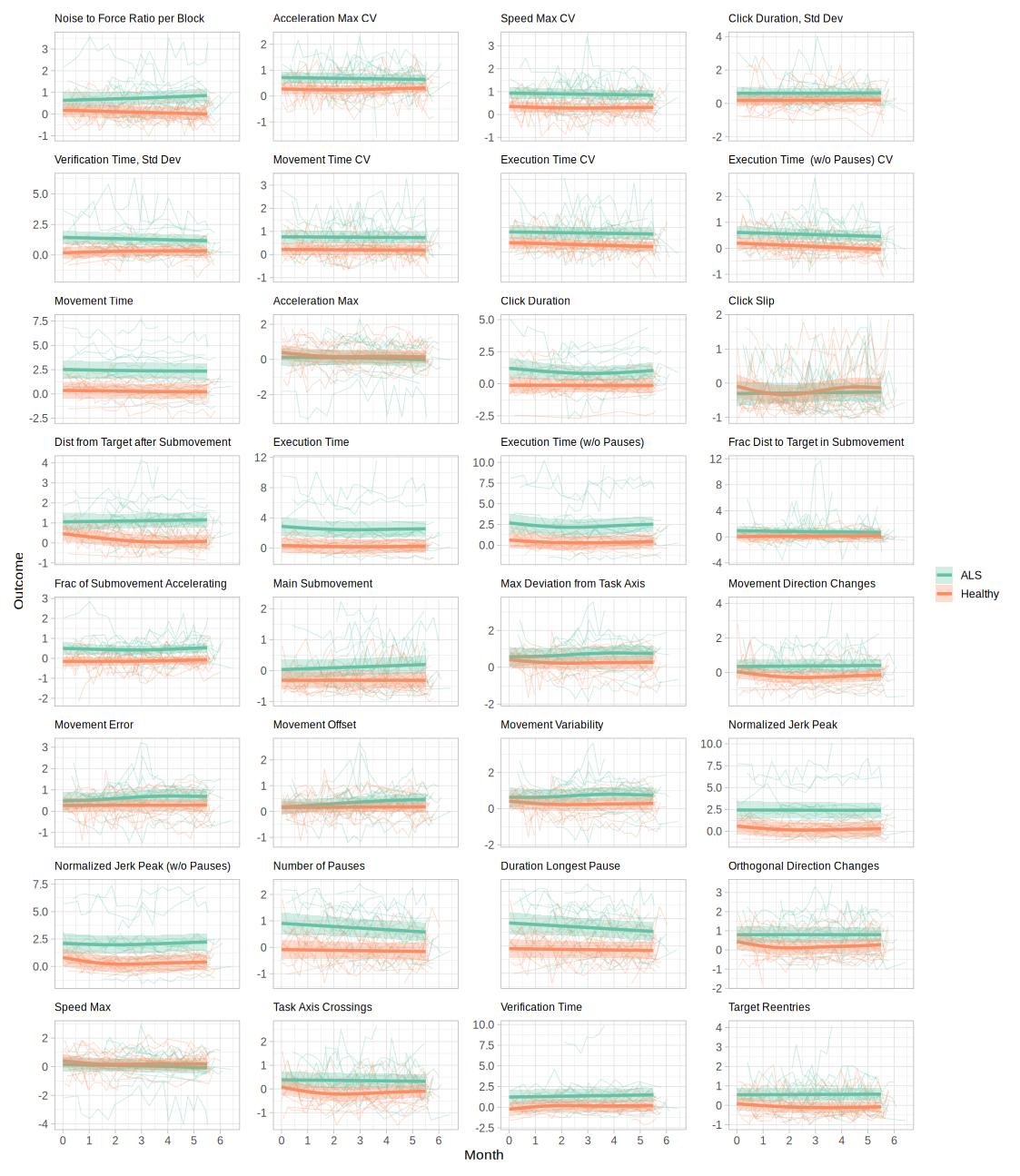


Supplementary Figure 3. Illustration of detrending process and effect on ICC estimates or speaking rate measure. Raw and detrended data are shown in (a). Linear fits for each participant's raw and detrended data are shown in (b), illustrating the removal of macro-level longitudinal patterns. (c) shows the within-participant and between-participant variance estimates, and resulting ICCs for raw and detrended data. In both healthy and ALS groups, the within-participant variability is substantially reduced after detrending, indicating the removal of the unwanted variability associated with longitudinal change.


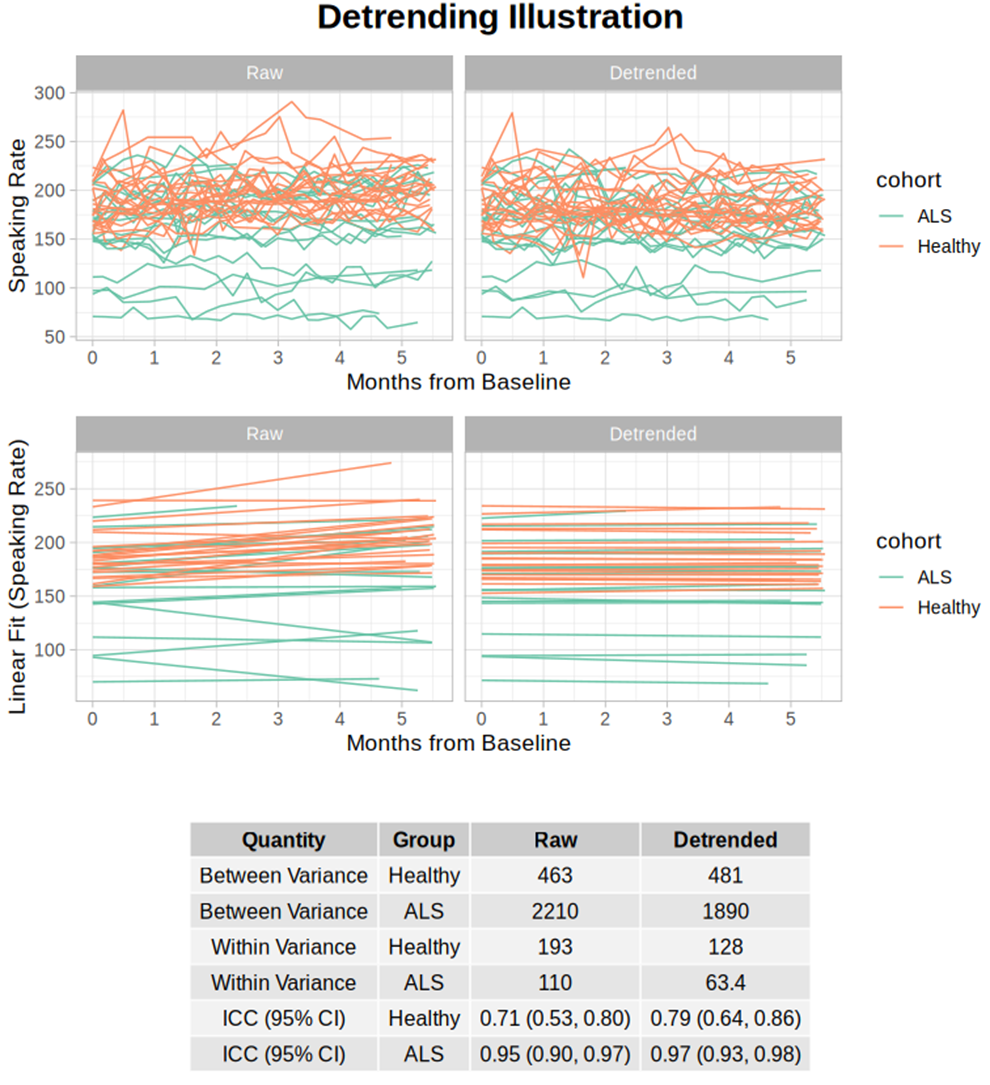

Supplement: Multimedia Appendix 1 [file formative-v10-e85142-s001.docx]
